# Evidencia de mundo real sobre adecuación farmacológica en diabetes tipo 2 y enfermedad cardiovascular

**DOI:** 10.31053/1853.0605.v80.n4.42272

**Published:** 2023-12-26

**Authors:** Carolina Pintos, Mariana Andrea Burgos, Natalia Inés Pasik, Sofía Piccioli, María Florencia Grande Ratti, María Paula Russo

**Affiliations:** 1 Departamento de Medicina Ambulatoria y Atención Primaria. Servicio de Clínica Médica, Hospital Italiano de Buenos Aires. Buenos Aires Argentina; 2 Área de Investigación en Medicina Interna, Hospital Italiano de Buenos Aires Buenos Aires Argentina; 3 CONICET (Consejo Nacional de Investigaciones Científicas y Técnicas) Buenos Aires Argentina; 4 Departamento de Medicina Interna. Servicio de Clínica Médica, Hospital Italiano de Buenos Aires

**Keywords:** diabetes mellitus tipo 2, enfermedades cardiovasculares, hipoglucemiantes, inhibidores del cotransportador de sodio-glucosa 2, liraglutida, diabetes mellitus, type 2, cardiovascular diseases, hypoglycemic agents, sodium-glucose transporter 2 inhibitors, liraglutide, diabetes mellitus tipo 2, doenças cardiovasculares, hipoglicemiantes, inibidores do transportador 2 de sódio-glicose, liraglutida

## Abstract

**Objetivo:**

Estimar la proporción de personas con Diabetes Mellitus tipo 2 (DM2) y Enfermedad Cardiovascular (ECV) establecida que reciben tratamiento farmacológico anti-diabético con evidencia de beneficio cardiovascular en un hospital en Argentina.

**Materiales y Métodos:**

Estudio de corte transversal realizado en el Hospital Italiano de Buenos Aires. Se incluyó una muestra consecutiva de pacientes adultos afiliados a prepaga institucional activos a Marzo 2020, con diagnóstico de DM2 y ECV establecida. Los datos se tomaron de la Historia Clínica Electrónica. Se informó la proporción de adecuación farmacológica (uso combinado de metformina más inhibidores del cotransportador de sodio glucosa tipo 2 y/o agonistas del Péptido Similar al Glucagón tipo 1) con su respectivo IC95%.

**Resultados:**

Se incluyeron 1539 pacientes, con una media de edad 76,2 años, 65,3% eran de sexo masculino, 81,6% con sobrepeso u obesidad. Un 74,9% de los pacientes tenían registro de hemoglobina glicosilada en el último año, con un valor promedio de 6,9% (DE 1,2). Las drogas más prescritas fueron: metformina (61,3%), insulina (26,7%), y gliptinas (11%). Del total de pacientes incluidos, 82 presentaron adecuación fármaco-terapéutica antidiabética, con una prevalencia de 5,3% (IC95% 4,2-6,5).

**Conclusiones:**

La prevalencia de prescripción de drogas antidiabéticas con evidencia de beneficio cardiovascular fue de 5,3% (IC95% 4,2-6,5). Esta información extraída de evidencia del mundo real identifica la baja frecuencia de prescripción de este tipo de fármacos al momento del estudio en una población de alto riesgo cardiovascular.

## Abstract (en)

**Purpose:**

To estimate the proportion of individuals with Type 2 Diabetes Mellitus (DM2) and established Cardiovascular Disease (CVD) receiving pharmacological anti-diabetic treatment with evidence of cardiovascular benefit at a hospital in Argentina.

**Materials and Methods:**

A cross-sectional study was conducted at the Hospital Italiano de Buenos Aires. A consecutive sample of adult patients affiliated with the institutional health plan as of March 2020, with a diagnosis of DM2 and established CVD, was included. Data were retrieved from the Electronic Health Record. The proportion of pharmacological adequacy (combined use of metformin plus sodium-glucose cotransporter-2 inhibitors and/or glucagon-like peptide-1 receptor agonists) with its respective 95% confidence interval (CI) was reported.

**Results:**

A total of 1539 patients were included, with a mean age of 76.2 years; 65.3% were male, and 81.6% were overweight or obese. Among the patients, 74.9% had records of glycosylated hemoglobin in the last year, with an average value of 6.9% (SD 1.2). The most prescribed drugs were metformin (61.3%), insulin (26.7%), and gliptins (11%). Out of the total included patients, 82 showed pharmacotherapeutic adequacy for diabetes, with a prevalence of 5.3% (95% CI 4.2-6.5).

**Conclusions:**

The prevalence of prescribing anti-diabetic drugs with evidence of cardiovascular benefit was 5.3% (95% CI 4.2-6.5). This real-world evidence highlights the low frequency of prescription of this type of medication at the time of the study in a high-risk cardiovascular population.

## Resumo

**Objetivo:**

Estimar a proporção de pessoas com Diabetes Mellitus tipo 2 (DM2) e Doença Cardiovascular (DCV) estabelecida que recebem tratamento farmacológico anti-diabético com evidência de benefício cardiovascular em um hospital na Argentina.

**Materiais e Métodos:**

Estudo transversal realizado no Hospital Italiano de Buenos Aires. Foi incluída uma amostra consecutiva de pacientes adultos afiliados a um plano de saúde institucional ativos em março de 2020, com diagnóstico de DM2 e DCV estabelecida. Os dados foram obtidos do Prontuário Eletrônico. Foi relatada a proporção de adequação farmacológica (uso combinado de metformina mais inibidores do cotransportador de sódio glicose tipo 2 e/ou agonistas do Peptídeo Similar ao Glucagon tipo 1) com o respectivo IC95%.

**Resultados:**

Foram incluídos 1539 pacientes, com média de idade de 76,2 anos, sendo 65,3% do sexo masculino e 81,6% com sobrepeso ou obesidade. Um total de 74,9% dos pacientes tinham registro de hemoglobina glicada no último ano, com valor médio de 6,9% (DE 1,2). As drogas mais prescritas foram: metformina (61,3%), insulina (26,7%) e gliptinas (11%). Do total de pacientes incluídos, 82 apresentaram adequação farmacoterapêutica antidiabética, com prevalência de 5,3% (IC95% 4,2-6,5).

**Conclusões:**

A prevalência de prescrição de drogas antidiabéticas com evidência de benefício cardiovascular foi de 5,3% (IC95% 4,2-6,5). Essa informação extraída de evidências do mundo real identifica a baixa frequência de prescrição desse tipo de fármaco no momento do estudo em uma população de alto risco cardiovascular.

**CONCEPTOS CLAVE**

Qué se sabe sobre el tema.

La diabetes mellitus tipo 2 representa un problema de salud pública a nivel global. Su tratamiento requiere de un enfoque integral con foco en la salud cardiovascular. A la fecha existen diversos fármacos con evidencia de beneficio cardiovascular con pocos datos sobre su uso en nuestro medio.

Qué aporta este trabajo.

Pese a la evidencia y las recomendaciones actuales, el porcentaje de adecuación farmacológica fue bajo en esta población con enfermedad cardiovascular establecida.

**Divulgación**

La diabetes mellitus tipo 2 representa uno de los principales factores de riesgo para el desarrollo de enfermedad cardiovascular deteriorando la calidad de vida y aumentando la mortalidad de quienes la padecen. Cerca del 13% de la población Argentina tiene diabetes, siendo la sexta causa de muerte en la región de las Américas.

Para su tratamiento es necesario un abordaje integral con eje central en la salud cardiovascular.

La utilización de fármacos con evidencia de beneficio cardiovascular parece verse limitada por diversos factores, entre ellos su elevado costo así como la inercia terapéutica de los profesionales a cargo del seguimiento de este grupo de pacientes. Entender las barreras que existen para el uso de estas drogas resulta de vital importancia para mejorar la sobrevida de las personas con diabetes.

## Introducción

La diabetes representa un problema de salud pública con gran impacto a nivel mundial ^1^ . En el año 2021, según datos de la International Diabetes Federation (IDF), 10,5% de la población mundial adulta tenía diagnóstico de diabetes, representando la diabetes de tipo 2 (DM2) el 90% de los casos ^2^ . De todos ellos, 62 millones habitaban la región de las Américas ^3^ . En Argentina, según la Cuarta Encuesta Nacional de Factores de Riesgo, la prevalencia por autorreporte de glucemia elevada y/o diabetes en la población mayor de 18 años de edad fue de 12,7% ^4^ .

La DM2 constituye un factor de riesgo para el desarrollo de enfermedad cardiovascular (ECV), siendo el accidente cerebrovascular y la cardiopatía isquémica las principales causas de morbimortalidad en esta población ^5, 6^ . Un metaanálisis realizado en 2018 informó una prevalencia global de ECV del 32,2% en pacientes con DM2 (7). En las Américas, la diabetes constituye la sexta causa de muerte y la segunda causa de años de vida perdidos por discapacidad, posicionándose como la región con mayor cantidad de años de vida saludable perdidos debido a la DM2 en todo el mundo ^3^ .

El tratamiento de las personas con DM2 requiere un enfoque multifactorial que incluya medidas tanto farmacológicas como no farmacológicas, con metas individualizadas de tratamiento. Numerosos estudios han demostrado que el foco debe estar centrado en el riesgo cardiovascular y en la presencia o no de ECV establecida ^8-11^ , independientemente del control glucémico. Sin embargo, el acceso desigual a los servicios de salud, en términos de tratamientos farmacológicos y tecnologías constituye una de las principales barreras para el diagnóstico, control y seguimiento de esta enfermedad ^12, 13^ .

Las recomendaciones nacionales e internacionales, basadas en el análisis de información proveniente de ensayos clínicos controlados (ECA), sugieren el uso de fármacos con evidencia de beneficio cardiovascular y renal en el grupo de pacientes con DM2 y ECV establecida o con alto riesgo de desarrollarla. La recomendación orienta al uso de los inhibidores del cotransportador sodio glucosa tipo 2 (iSGLT2) y agonistas del péptido similar al glucagón tipo 1 (aGLP1) ^9-11^ .

Sin embargo, la evidencia obtenida de los ECA tiene una validez externa limitada, muchas veces no aplicable a pacientes en la práctica clínica habitual. De allí surge la necesidad de complementar tal información con datos provenientes del mundo real (evidencia del mundo real o "RWE" por sus siglas en inglés) y así respaldar la seguridad y/o eficacia de los fármacos estudiados ^14^ .

En Argentina, según evidencia reciente, la utilización de medicamentos antidiabéticos con protección cardiovascular comprobada en población con DM2 y ECV establecida es baja (11,5%) ^15^ . En nuestra población se desconoce el porcentaje de pacientes con tales características que reciben tratamiento según lineamientos nacionales e internacionales ^1,8, 16^ .

Nuestro objetivo fue estimar la proporción de pacientes con diagnóstico de DM 2 y ECV establecida que reciben tratamiento farmacológico antidiabético adecuado que incluya drogas con beneficio cardiovascular demostrado, así como describir las características clínico epidemiológicas de esa población.

## Materiales y métodos

Se realizó un estudio de corte transversal en el Hospital Italiano de Buenos Aires, Argentina, que incluyó pacientes adultos afiliados a prepaga institucional.

El número de socios en el padrón del Plan de Salud varía mes a mes debido a ingresos y egresos, por lo que se definió al total de afiliados activos a la fecha del 1 de Marzo 2020 como denominador para este estudio. Se incluyeron específicamente aquellos mayores de 18 años con diagnóstico previo de DM2, cuando el mismo constara como problema registrado en la Historia Clínica Electrónica (HCE), y que tuvieran antecedente de ECV establecida. Esta última se definió como la presencia de enfermedad coronaria (infarto de miocardio o isquemia miocárdica con o sin requerimiento de revascularización, angina estable), enfermedad cerebrovascular (ictus isquémico o ataque isquémico transitorio) y/o enfermedad arterial periférica. Se excluyeron pacientes embarazadas con DM2 por contraindicaciones farmacológicas para este grupo.

Los afiliados a PS se comportan como una cohorte cerrada, de la cual se dispone de información confiable sobre sus características demográficas, las prescripciones realizadas por los médicos de la institución y dispensación de medicamentos de farmacia. La fuente de recolección de datos fue la HCE que funciona como único repositorio centralizado de datos informatizados.

Se definieron las siguientes variables principales, basadas en los registros de los últimos 12 meses (previo al 1/03/2020):

-Adecuación fármaco-terapéutica antidiabética: Prescripción de fármacos antidiabéticos que incluyeran metformina más adicional de iSGLT2 y/o aGLP1.

-Tratamiento de prevención cardiovascular secundaria: Prescripción de antiagregante plaquetario (AAS), más terapia antihipertensiva con inhibidor de la enzima convertidora de angiotensina (IECA) o agonista del receptor de aldosterona II (ARA II), más terapia hipolipemiante (estatinas).

-Tratamiento adecuado: Constructo definido como adecuación fármaco-terapéutica antidiabética más el tratamiento cardiovascular completo de prevención secundaria.

Otras variables fueron: demográficas (edad, sexo), clínicas (obesidad, hipertensión arterial, dislipemia, enfermedad renal crónica, depresión), bioquímicas (HbA1c, perfil lipídico).

Para estimar la prevalencia de pacientes con adecuación fármaco-terapéutica antidiabética se consideró como denominador a toda la población con DM2 y ECV establecida, y como numerador a quienes se les prescribió metformina más iSGLT-2 y/o a-GLP1. Se reportaron resultados como proporción con su respectivo intervalo de confianza del 95%. Para responder a los objetivos secundarios se utilizó estadística descriptiva. Las variables cuantitativas se expresaron en media y desvío estándar o mediana e intervalo intercuartílico 25-75 según distribución y las variables categóricas se expresaron en frecuencia absoluta y relativa (porcentaje) con sus respectivos intervalos de confianza (IC95%). El análisis estadístico se realizó con software STATA 17.

Se contó con la aprobación del comité de ética local (CEPI#6205), y todos los datos del estudio fueron tratados con máxima confidencialidad.

## Resultados

**Figure 1:**
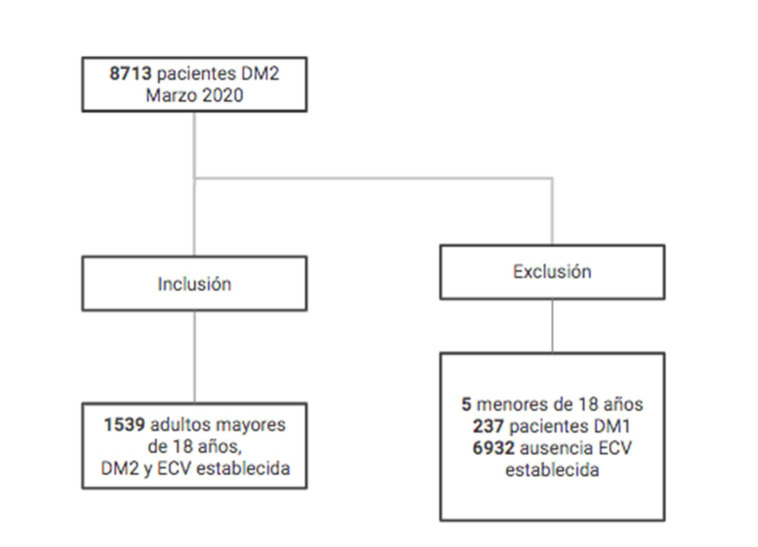
Diagrama de flujo de los pacientes incluidos.

De 8713 personas con antecedente de diabetes, se incluyeron 1539 adultos con diagnóstico de DM2 y ECV establecida, representando el 17,7% (IC95% 16,9-18,5). En la Figura 1 se muestra el diagrama de flujo de los pacientes incluidos. La prevalencia de pacientes con adecuación fármaco-terapéutica antidiabética fue de 5,3% (IC95% 4,2 - 6,5).

En cuanto a las características basales de la población, la media de edad fue de 76,2 años (DE+/-10,5), 65,3% sexo masculino, 81,6% con sobrepeso u obesidad. El 75% de la población en estudio tuvo al menos una medición de hemoglobina glicosilada (HbA1c) en el último año, con una media de 6,9% (DE +/- 1,2). Respecto a las complicaciones macrovasculares, el 61,5% tenían como antecedente infarto de miocardio o enfermedad coronaria, 40,6% accidente cerebrovascular y 12,7% enfermedad vascular periférica. Las características generales de los participantes se detallan en la Tabla 1.

**Tabla 1:** Descripción de las características basales de la población.

Características basales	n 1539
Edad, en años *	76,2 (DE+/-10,5)
Sexo Masculino %(n)	65,3 (1005) IC95% (62,9 - 67,7)
IMC*	29,7 (DE +/- 5)
Sobrepeso %(n)	39,5 (608) IC95%(37,1 - 42)
Obesidad %(n)	42,1 ( 648) IC95%(39,6 - 44,6)
Complicaciones macrovasculares	
IM / Enf coronaria %(n)	61,5 (947) IC95%(59,1 - 64)
ACV %(n)	40,6 (625) IC95%(38,1 - 43,1)
EVP %(n)	12,7 (195) IC95%(11 - 14,4)
Otras complicaciones	
Pie diabetico %(n)	18,3(282) IC95%(16,4 - 20,3)
Retinopatía %(n)	4,4 (67) IC95%(3,9 - 5,5)
Nefropatía %(n)	1,7 (26) IC95%(1,1 - 2,5)
Comorbilidades cardiovasculares	
HTA %(n)	89,4 (1376) IC95%(87,8 - 90,9)
Tabaquismo %(n)	51,3 (790) IC95%(48,8 - 53,9)
Dislipemia %(n)	20,5 (316) IC95%(18,5 - 22,6)
ERC %(n)	15,8 (235) IC95%(13,5 - 17,2)
IC %(n)	6,8 (105) IC95%(5,6 - 8,2)
Hemodiálisis %(n)	0,3 ( 5) IC95%(0,1 - 0,8)
Otras comorbilidades	
Depresión %(n)	21 (323) IC95%(19 - 23,1)
Deterioro cognitivo %(n)	9,2 (141) IC95%(7,8 - 10,7)
Demencia %(n)	4,9 (76) IC95%(3,9 - 6,1)
Apnea de sueño %(n)	4,2 (64) IC95%(3,2 - 5,3)
Laboratorio en el último año	
HbA1c medida	74,9 (1153) IC95%(72,7 - 77,1)
HbA1c, valor (%)	6,9 (DE +/- 1,2)
Clearance Creatinina medido %(n)	25,2 (387) IC95%(23 - 27,4)
Clearance Creatinina. valor	43,6 (DE +/- 11,6)
HDL, medida %(n)	74,5 (1151) IC95%(72,5 - 77)
Valor (mg/dL)	43,3 (DE +/- 11,5)
LDL %(n)	72,8 (1120) IC95%(70,5 - 75)
Valor (mg/dL)	78,6 (DE +/- 31,6)
TAG %(n)	74,1 (1140) IC95%(71,8 - 76,2)
Valor (mg/dL)	148,9 (DE +/- 88)

ACV: accidente cerebrovascular; ERC enfermedad renal crónica; EVP: enfermedad vascular periférica; HbA1c: hemoglobina glucosilada; HTA: hipertensión arterial; IMC: índice de masa corporal; IM: infarto de miocardio; IC insuficiencia cardíaca; TAG triglicéridos

*Media y Desvío estándar

En cuanto al tratamiento farmacológico, se observó que 61,3% recibía terapia antidiabética con metformina, mientras que un 4% y un 2,4% tenían prescritos fármacos de los tipos aGLP1 e iSGLT2 respectivamente. El porcentaje de prevalencia de tratamiento de prevención cardiovascular secundaria completo observado fue de 36,7% (IC95% 34,4 - 39,2).

En relación a quienes recibieron tratamiento adecuado (adecuación fármaco-terapéutica antidiabética más tratamiento cardiovascular de prevención secundaria) el porcentaje fue sólo de 2,9% (IC95% 2,1- 3,9). La información detallada se muestra en la Tabla 2.

**Tabla 2:** Características del tratamiento farmacológico

Tratamiento farmacológico (n 1539)	
Antidiabéticos	
Metformina %(n)	61,3(944) IC95%( 58,9 - 63,8)
Pioglitazona %(n)	2,7(42) IC95%(2 - 3,7)
Sulfonilureas %(n)	2,5 (39) IC95%(1,8 - 3,4)
Metiglinidas (glinidas) %(n)	0,9 (14) IC95%(0,04 - 0,2)
iDPP4 (gliptinas) %(n)	11 (169) IC95%(9,5 - 12,7)
aGLP1 (Liraglutida) %(n)	4 (62) IC95%(3,1 - 5,1)
iSGLT2 %(n)	2,4 (37) IC95%(1,7 - 3,3)
Insulina %(n)	26,6 (410) IC95%(17 - 33)
Antiagregantes	
AAS %(n)	59,8 (920) IC95%(57,3 - 62,2)
Antihipertensivos	
Beta bloqueantes %(n)	62,3 (958) IC95%(59,8 - 64,7)
IECA %(n)	36,8(566) IC95%(34,4 - 39,2)
ARA II %(n)	28 (431) IC95%(25,8 - 30,3)
Bloqueantes Cálcicos %(n)	37,3 (574) IC95%(34,9 - 39,8)
Diuréticos tiazídicos %(n)	18,8 (290) IC95%(16,9 - 20,9)
Diuréticos asa (furosemida) %(n)	16,4 (253) IC95%(14,6 - 18,4)
Diuréticos ahorradores de potasio %(n)	5,5 (84) IC95%(4,4 - 6,7)
Hipolipemiantes	
Estatinas %(n)	72,4 (1114) IC95%(70,1 - 74,6)
Fibratos %(n)	5,1 (78) IC95%(4 - 6,3)
Ezetimibe %(n)	13,6 (209) IC95%(11,9 - 15,4)

aGLP1: agonistas del péptido similar al glucagon tipo 1; ARA II: antagonistas del receptor angiontensina 2; AAS: ácido acetil salicílico; iSGLT2 inhibidores del cotransportador sodio glucosatipo 2; iDPP4: inihibidores de dipeptidasa 4

## Discusión

En nuestro estudio la prescripción de fármacos con beneficio cardiovascular demostrado fue del 5,3%, cifra menor respecto a resultados de estudios similares realizados en nuestro país. Entre ellos se destacan el estudio CAPTURE que mostró una prescripción de 12,8 % para el mismo grupo de fármacos ^15^ y el estudio de Forte y col. ^17^ que informó una prescripción del 15%.

En un análisis más detallado, observamos que la prescripción fue 4% en el caso de los aGLP1, cifra similar respecto al resto de los trabajos mencionados, mientras para el grupo iSGLT2 fue de 2,4%, inferior al estudio CAPTURE (8,1%) y al trabajo de Forte y col. (12,8%) ^15, 17^ .

La baja prevalencia de prescripción podría atribuirse a dos factores principales: el alto costo de estos fármacos , lo que dificulta el acceso a los mismos debido a que la cobertura es parcial por parte de los prestadores de salud, y la inercia terapéutica por parte de los profesionales que conlleva a una demora en el inicio o en la intensificación de tratamientos cuando están indicados. Entre otros factores a considerar se encuentran la contraindicación de presciprción de fármacos como la metformina y los iSLGT2 por falla renal en estadío dialítico o tasa de filtración marcadamente disminuída.

Respecto al tratamiento de prevención cardiovascular secundaria, el 72,4% de la población analizada en el presente trabajo tenían prescritos fármacos hipolipemiantes, 64,8% terapia antihipertensiva y 59,8% terapia antiagregante, mientras en el estudio CAPTURE Argentina las cifras reportadas fueron de 71,5%, 87,9% y 66,9% para mismo grupos de drogas respectivamente ^15^ . Pese a la diferencias observadas, ambos trabajos demostraron menor proporción de indicación en comparación con lo reportado por Forte y col. en el que el 81,5% de la población incluida tenía prescrito tratamiento hipolipemiante, el 87,8% terapia antihipertensiva y 72,9% tratamiento antiagregante ^17^ .

Cabe destacar que en el caso del estudio realizado por Forte y col. todos los pacientes incluidos recibían atención por parte de médicos cardiólogos, lo cual podría estar en relación con la mayor prescripción de drogas de tratamiento de prevención cardiovascular secundaria ^17^ . Para el caso del estudio CAPTURE Argentina, los pacientes incluidos recibían atención en consultorio de médicos generalistas y/o diabetólogos y endocrinólogos ^15^ . Respecto al presente trabajo, no se conocen datos sobre la especialidad de los médicos que realizaban el seguimiento de los pacientes pero la sospecha es que hay muchos de atencion primaria dada la idiosincracia de atencion.

En cuanto a la terapia antidiabética, el fármaco más utilizado, tanto en monoterapia como en terapia combinada, fue la metformina con una frecuencia de prescripción del 61,3% en el presente trabajo y 81,2% en el caso del trabajo de Forte ^17^ . Para el caso de tratamiento con insulina, el 26,7% de nuestros paciente tenían indicado el mismo, con una prevalencia comparable para el caso del estudio de Forte ^17^ , mientras en el estudio CAPTURE Argentina se informó una prevalencia de indicación del 43,6% sin reportar datos respecto a la prescripción de metformina ^15^ .

En referencia al control metabólico, el 74,9% de los pacientes tenían al menos una medición de HbA1c en el último año, con una media de 6,9%, cifra similar a la informada por los otros estudios (7,2% CAPTURE; 7,3% Forte y col.) ^15, 17^ .

La forma de presentación de ECV más frecuente fue la enfermedad coronaria tanto en el presente estudio como en CAPTURE Argentina y el trabajo realizado por Forte y col. con una prevalencia estimada de 61,7%, 20,2% y 34% respectivamente ^15, 17^ , seguida en frecuencia por ACV entre los pacientes de nuestro centro con una prevalencia de 40,6% cifra significativamente mayor en comparación con el 8,3% y 4% reportados en CAPTURE Argentina y el trabajo de Forte y col. respectivamente ^15, 17^ .

En relación a las características de la población, en todos los estudios mencionados la mayor parte de los pacientes eran de sexo masculino, siendo la media de edad de 76,2 años en el caso de nuestro trabajo, aproximadamente 10 años más que en el resto de los estudios.

Según una revisión sobre prevalencia de diabetes en América Latina ^18^ la pirámide poblacional se ha desplazado hacia una mayor proporción de adultos mayores afectados, lo que se acompaña de mayor complejidad en la atención sanitaria y fragilidad, viéndose esto reflejado en la media de edad de los pacientes incluídos en el presente estudio.

La comorbilidad más frecuente en todos los casos fue la hipertensión arterial (entre un 80 y un 93% de la población analizada) seguida por obesidad y dislipemia. Como factor de riesgo asociado al desarrollo de enfermedad cardiovascular, el 51,3% de nuestros pacientes tenían como antecedente el tabaquismo (actual o pasado), 36,7% en el caso de las personas incluídas en el estudio CAPTURE Argentina ^15^ y sólo el 6,8% de los pacientes con ECV establecida incluidos en el trabajo de Forte y col ^17^ .

El presente trabajo permitió visibilizar la brecha existente entre las recomendaciones internacionales actuales y la práctica clínica real en nuestro medio. La evaluación de potenciales barreras y facilitadores para superar esta diferencia podría dar lugar a beneficios en el pronóstico de estos pacientes.

La implementación de un abordaje integral más allá del control glucémico, el desafío de la inercia terapéutica, y la utilización de estrategias con beneficios cardiovasculares comprobados oportunamente podrían representar herramientas para mejorar el pronóstico de esta creciente población de pacientes.

Reconocemos que existen ciertas limitaciones. En primer lugar, fue realizado en un único centro médico perteneciente al ámbito de la medicina privada, lo que podría introducir un sesgo de selección y limitar su validez externa. En segundo lugar, el análisis de datos se realizó de manera retrospectiva, lo que conlleva un eventual subregistro (sesgo de información) y pueden no estar disponibles todas las variables de interés y confundidoras (ej: fecha de última consulta realizada por el paciente). En este sentido, ese dato resulta relevante para la prescripción farmacológica, ya que la decisión en cuanto a inicio o cambio en el esquema de tratamiento suele requerir más de un encuentro con el paciente. Asimismo, la ventana temporal del muestreo también puede estar sujeto a sesgos de información debido a que, para el año 2019, la introducción de iSGLT2 y/o aGLP1 era reciente. Esto podría explicar la baja prescripción farmacológica, y entonces futuros estudios serán necesarios para explorar la indicación terapéutica a lo largo del tiempo.

Pese a esto, entendemos que los datos tienen gran validez interna y son relevantes para la mejora en la atención de los pacientes.

Mientras tanto, consideramos como fortalezas el gran tamaño muestral respecto a otros estudios realizados en nuestro país. Destacamos también la calidad de los datos obtenidos de una fuente confiable como es la historia clínica electrónica con pocos datos perdidos.

Por otro lado, el presente estudio no contó con financiamiento de la industria.

## Conclusión

La tasa de prescripción de fármacos con beneficio cardiovascular demostrado entre los pacientes con antecedentes de DM2 y ECV establecida fue baja.

Consideramos que se requieren de más estudios que permitan explorar las barreras en la implementación de recomendaciones con el fin de modificar la forma de prescribir de los profesionales en pos de brindar la mejor estrategia de tratamiento disponible para esta población.
